# Five-year outcomes following left ventricular assist device implantation in England

**DOI:** 10.1136/openhrt-2021-001658

**Published:** 2021-05-11

**Authors:** Alex Bottle, Puji Faitna, Paul P Aylin, Martin R Cowie

**Affiliations:** 1 School of Public Health, Faculty of Medicine, Imperial College London, London, UK; 2 General Practice and Public Health, Imperial College London, London, UK; 3 School of Cardiovascular Medicine & Sciences, Faculty of Life Sciences & Medicine, King's College London, London, UK

**Keywords:** heart failure, left ventricular assist device, outcome assessment, health care

## Abstract

**Objective:**

Implant rates of mechanical circulatory supports such as left ventricular assist devices (LVAD) have steadily increased in the last decade. We assessed the utility of administrative data to provide information on hospital use and outcomes.

**Methods:**

Using 2 years of national hospital administrative data for England linked to the death register, we identified all patients with an LVAD and extracted hospital activity for 5 years before and after the LVAD implantation date.

**Results:**

In the two index years April 2011 to March 2013, 157 patients had an LVAD implanted. The mean age was 50.9 (SD 15.4), and 78.3% were men. After 5 years, 92 (58.6%) had died; the recorded cause of death was noncardiovascular in 67.4%. 42 (26.8%) patients received a heart±lung transplantation. Compared with the 12 months before implantation, the 12 months after but not including the month of implantation saw falls in total inpatient and day case admissions, a fall in admissions for heart failure (HF), a rise in non-HF admissions, a fall in emergency department visits not ending in admission and a rise in outpatient appointments (all per patient at risk). Postimplantation complications were common in the subsequent 5 years: 26.1% had a stroke, 23.6% had a device infection and 13.4% had a new LVAD implanted.

**Conclusions:**

Despite patients’ young age, their mortality is high and their hospital use and complications are common in the 5 years following LVAD implantation. Administrative data provide important information on resource use in this patient group.

**Key questions**

**What is already known about this subject?**

- Implant rates of mechanical circulatory supports such as left ventricular assist devices (LVAD) have steadily increased in the last decade. Most studies do not report outcomes beyond 2 years, and much information comes from trials, which may not be generalisable.

**What does this study add?**

- Despite patients’ young age, their mortality is high and their hospital use and complications are common in the 5 years following LVAD implantation. Administrative data provide important information on resource use in this patient group that can be more timely than that from registries.

**How might this impact on clinical practice?**

- National data sets can help inform assessment of outcome and healthcare utilisation for advanced heart failure therapies such as LVAD.

## Introduction

Heart failure (HF) affects 26 million people worldwide, and its incidence is growing in the UK.^1–3^ HF is expensive to treat and places a significant burden on healthcare systems.^3 4^ Improvements in HF treatments have meant that a growing number of patients live to develop severe HF, and, for these patients, heart transplants remain the gold standard treatment.^5 6^ Unfortunately, there is a worldwide shortage of heart donors, highlighting the practical limitations of heart transplants.^6 7^ Consequently, there has been a rise in mechanical circulatory support (MCS) implants to help bridge this gap.^8 9^

MCS is a good option for refractory patients with HF who are ineligible for heart transplants.^10 11^ Implant rates of MCS such as left ventricular assist devices (LVAD) have steadily increased in the last decade, offering improvements to the survival and quality of life of patients.^5 9 12 13^

Many studies assessing LVAD outcomes use randomised control trials (RCT), which may not be generalisable due to restrictive RCT selection criteria.^14^ Survival estimates following an LVAD implant range from 56% to 87% at 1 year and 43% to 84% at 2 years; most do not report survival rates beyond 2 years.^7 9 10^ More LVAD studies are needed to assess longer term survival, costs and complications, as this has limited cost-effectiveness evaluations.^15^ Administrative data can offer insights on longer term outcomes from a broader population as it has national coverage.^14^ The National Institute for Health and Care Excellence recommends using existing real-world data to inform decision-making.^16^ This study is the first to use English hospital administrative data, Hospital Episode Statistics (HES), to explore the longer term survival rates, complications and hospital service use following LVAD implantation.

## Materials and methods

### Data

We extracted records from England’s national hospital administrative database, HES, which comprises over 125 million admitted patient, outpatient and emergency department (ED) records from the National Health Service (NHS) annually. Submission is mandatory. Inpatient and day case diagnostic coding uses ICD-10 (International Statistical Classification of Diseases and Related Health Problems 10th Revision), but ED records use a much broader and more symptom-based approach. Each admission is assigned a primary ICD-10 diagnostic code by trained staff who determine this to be the primary reason why the patient is being treated; 19 secondary ICD-10 codes relate to comorbidities or complications during the admission. Up to 24 procedures are coded using the UK’s Office of Population Censuses and Surveys (OPCS) system. With a time-lag, the national HES database is linked to the national deaths registry, maintained by the Office of National Statistics, thereby capturing the date and causes of all deaths, including out-of-hospital deaths. We used HES data from April 2006 to March 2018, with linked death data to July 2018; for ED records reliable data only existed from April 2009.

### Cohort and outcomes

Using 2 years of HES, April 2011 to March 2013, we defined a cohort based on the inpatient admission record covering the implantation of each patient’s first device during the 2 years: the procedure date for this was their ‘index date’. An OPCS procedure code of K54.1 (‘open implantation of ventricular assist device’) in any position was used for the LVAD implantation. Patients with records with codes for the implantation or removal of such devices in the previous 5 years were excluded (K54.2 is the code for removal). Comorbidities were derived from the index admission and any admission in the previous year.

Total mortality, hospital activity by sector—outpatient clinic, ED, day case and inpatient admission—were the main outcomes. We divided inpatient and day case activity into that for HF (ICD-10 I50) and that for any non-HF conditions using the primary diagnosis field. We identified postimplantation admissions for removal of the device, implantation of a new LVAD, haemorrhagic stroke and ischaemic stroke (and all types combined). ICD-10 codes for the complications are given in the online supplemental appendix. Heart or heart and lung transplantation procedures were identified using OPCS codes K01 and K02. For all these outcomes, all consultant episodes within the admission and all diagnosis and procedure fields were examined.

10.1136/openhrt-2021-001658.supp1 Supplementary data

### Analysis

A Kaplan-Meier plot described the 5-year mortality and median survival since the index date. For hospital activity and reference costs, we calculated the monthly rates per patient at risk, that is, per patient still alive, for five years before and after the index month, giving 121 months (periods of 30 days) in total. Using the Aalen-Johansen method to handle competing risks, we plotted the proportions of patients over time on support, with a transplant or explanted (with the device removed). SAS V.9.4 was used for all analyses.

### Ethics

We have the approval from the Secretary of State and the Health Research Authority under Regulation 5 of the Health Service (Control of Patient Information) Regulations 2002 to hold confidential data and analyse them for research purposes (CAG ref 15/CAG/0005). We have approval to use them for research and measuring quality of delivery of healthcare, from the London—South East Ethics Committee (REC ref 15/LO/0824). We attest our strict compliance with the ISHLT ethics statement.

### Patient and public involvement

Patients or the public were not involved in the design, or conduct, or reporting or dissemination plans of our research.

## Results

### Patient characteristics

For the two index years from April 2011 to March 2013, there were 157 patients with an LVAD implanted. No patients were excluded due to missing or invalid data. The mean age was 50.9, and more than three-quarters were men. Patients had a median of three comorbidities listed in table 1 (IQR 2–5).

**Table 1:** Patient characteristics on admission for the LVAD implantation

Factor	N (% of total)
All patients	157
Age 18–39	34 (21.7)
Age 40–64	92 (58.6)
Age 65–74	22 (14.0)
Age 75–84	8 (5.1)
Age 85+	1 (0.6)
Age: mean (SD)	50.9 (15.4)
Women	34 (21.7)
Men	123 (78.3)
Coronary artery bypass graft	29 (18.5)
Percutaneous coronary intervention	19 (12.1)
Diabetes	24 (15.3)
Stroke	14 (8.9)
Pneumonia	22 (14.0)
Ischaemic heart disease	92 (58.6)
Atrial fibrillation	74 (47.1)
Valvular disorders	99 (63.1)
Hypertension	61 (38.9)
Peripheral vascular disease	37 (23.6)
Chronic pulmonary disease	29 (18.5)
Renal disease	45 (28.7)
Obesity	16 (10.2)
Dementia	0 (0)
Depression	7 (4.5)
Other mental health condition	27 (17.2)

LVAD, left ventricular assist device.

### Overall survival and time to other outcomes

**Figure 1:**
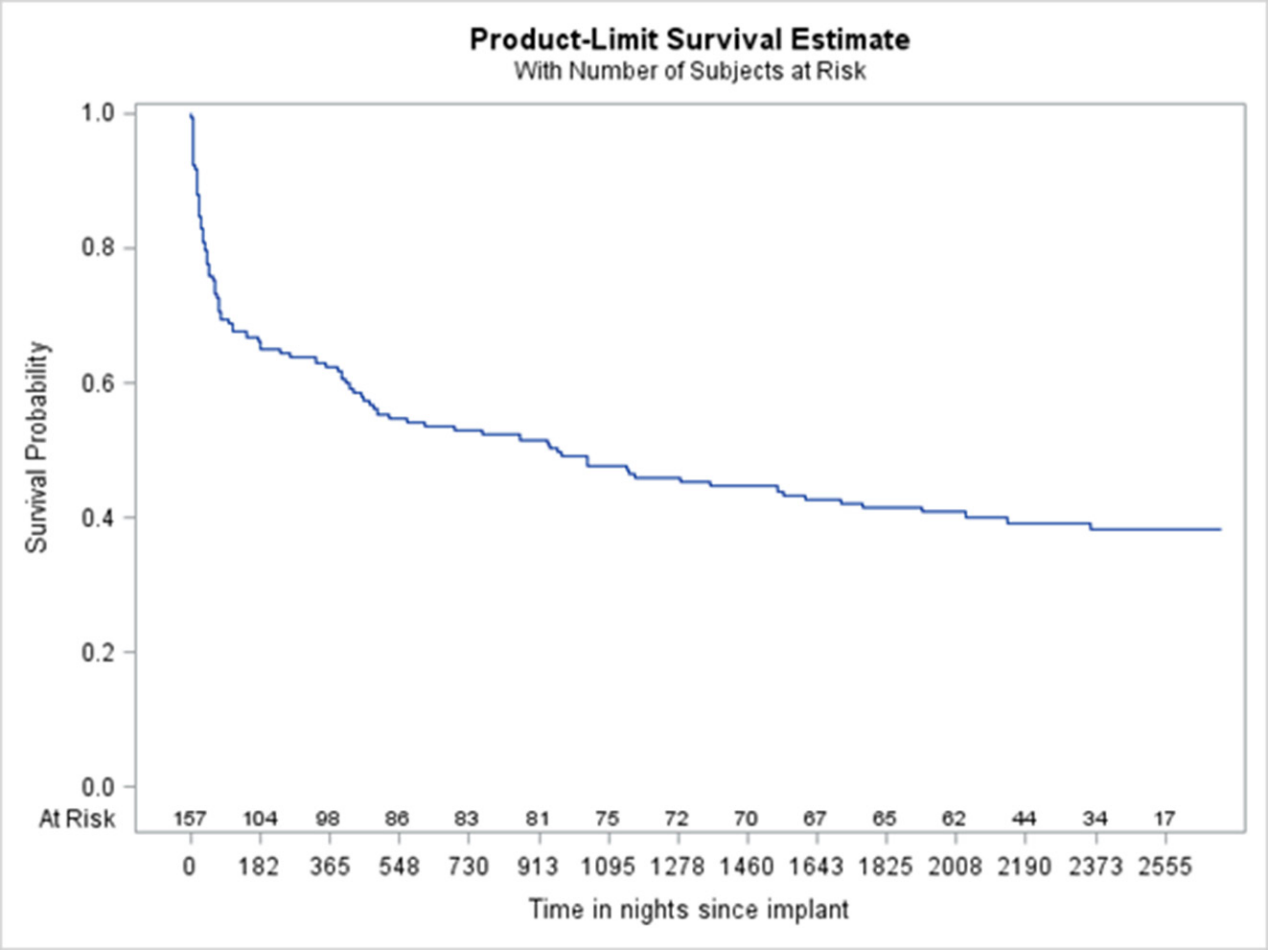
Kaplan-Meier plot of 5-year mortality following LVADs implanted between 1 April 2011 and 31 March 2013, plotted as days since implantation date. LVAD, left ventricular assist device.

By 5 years after the index date, 92 patients had died, for an all-cause case fatality rate of 58.6%; the 1-year rate was 39.5%, the 2-year rate was 47.8%. Figure 1 shows the Kaplan-Meier plot. Median survival was 960 days (2.6 years). HF was given as the main cause for 12.4% of deaths; other cardiovascular but non-HF causes accounted for 20.2%, with the remaining 67.4% noncardiovascular and non-HF.

During the 5 years’ follow-up, heart±lung transplantation was performed in 42 patients (26.8%) including during the index admission, with 14 deaths; excluding the index admission, there were 30 transplants with seven deaths. The median acute hospital length of stay (LOS) for the index stay was 24 nights (IQR 9–49); including any interhospital transfers gave a median LOS of 40 nights (IQR 23–70).

The median time to renewal in the 21 patients who had a second LVAD was 135 days (IQR 8–463).

The median time on support was 351 days (IQR 13–1171), estimated using the Kaplan-Meier method and defining transplant, explant or death as the end of support.

The median time to transplant in the 42 patients who had one was 433 days (IQR 82–900).

**Figure 2:**
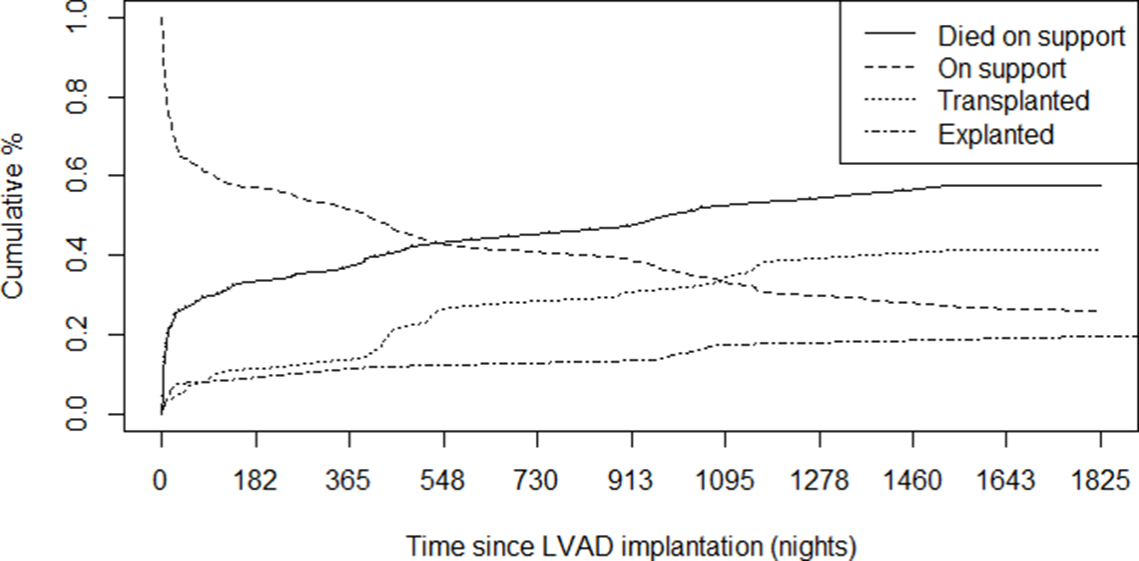
Cumulative incidence over 5 years of transplants, death and explants following LVADs implanted between 1 April 2011 and 31 March 2013. LVAD, left ventricular assist device.

Figure 2 shows the cumulative incidence of transplant, death and explant for all 157 patients.

### Hospital activity in the 5 years before and 5 years after implantation

**Figure 3:**
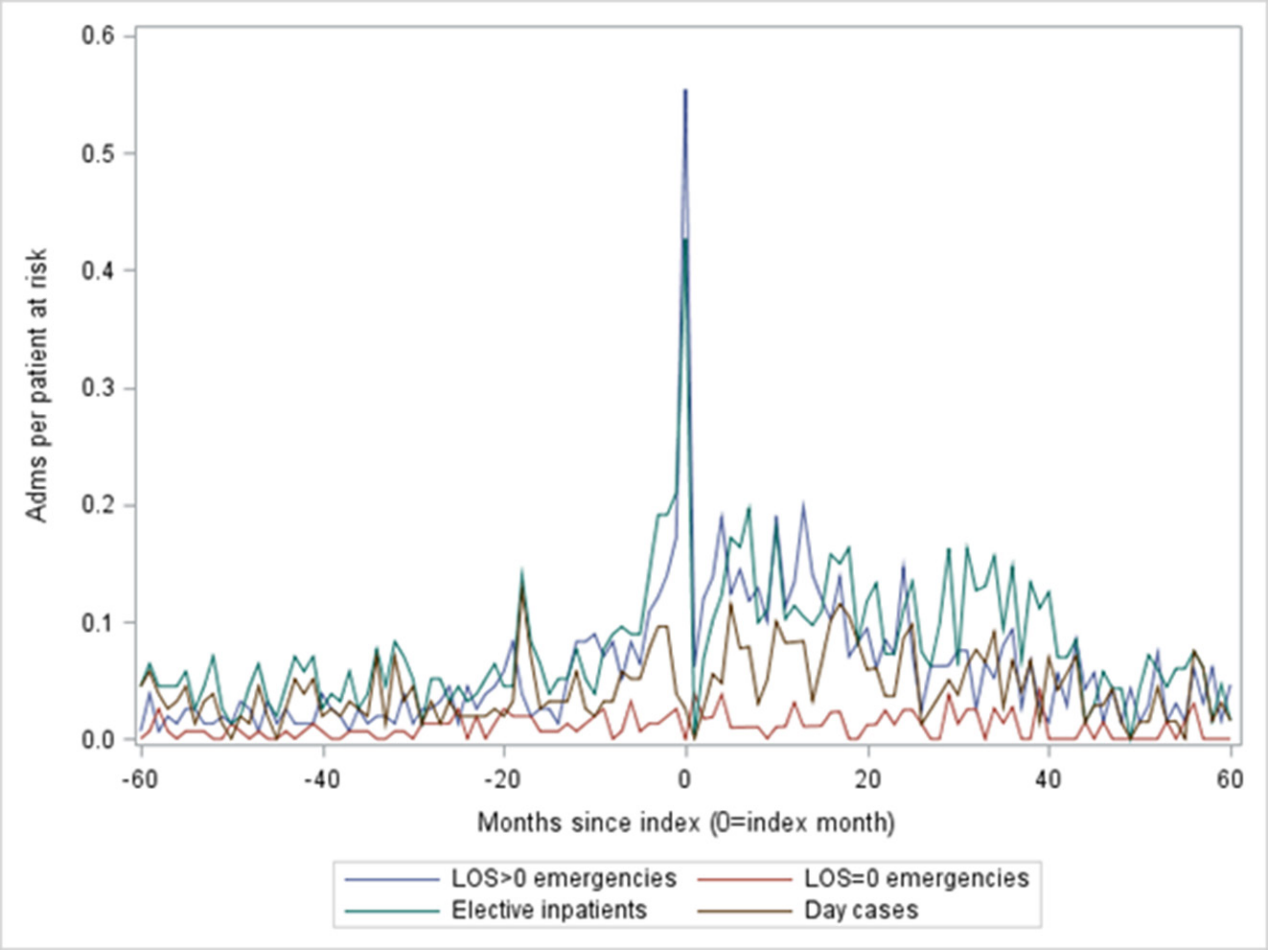
Admissions before and after LVAD implantation by type. LVAD, left ventricular assist device.

**Figure 4:**
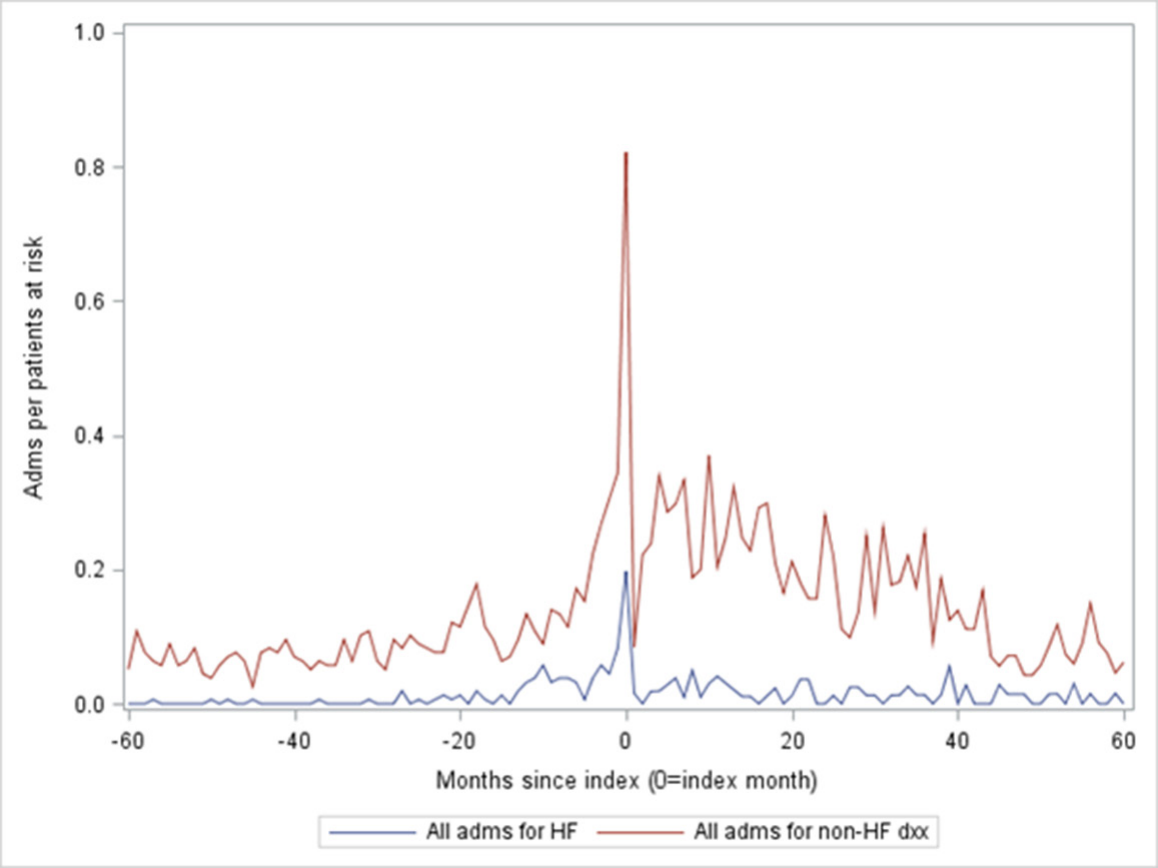
All admissions per person at risk, split by the primary diagnosis into HF (lower line) vs non-HF (upper line). HF, heart failure.

Figure 3 shows hospital admissions, expressed as admissions per patient at risk. The unplanned admissions with LOS >0 days (ie, at least one night) dominated for the index admission and were the most common type of admission both before and after the index date. In figure 4, all admissions (elective inpatient admissions, day cases and emergency inpatient admissions) have been split by the coded primary diagnosis into two groups: HF and non-HF admissions. HF admissions fell and non-HF admissions rose after implantation (table 2).

**Table 2:** Total inpatient and day case admissions by primary diagnosis in the 12 months before and the 12 months after the index month (index month not included)

	12 m before index	12 m after index
All admissions	421	344
Admissions per patient at risk	0.223	0.271
HF admissions	78	30
Admissions per patient at risk	0.0414	0.0236
Non-HF admissions	343	314
Admissions per patient at risk	0.1821	0.2475

HF, heart failure.

### Activity and costs in the year before and after the index month

The peak in ED visits not ending in admission occurred in the month before the index month. Mean outpatient appointments per patient at risk rose after the index month (table 3).

**Table 3:** Total ED visits not ending in admission and total outpatient department appointments for each group in the 12 months before and the 12 months after the index month (index month not included)

Activity	12 m before index	12 m after index
Total ED visits not ending in admission	80	53
Visits per patient at risk	0.059	0.051
Total cost (£)	142 702	222 405
Appointments per patient at risk	0.75	1.64

ED, emergency department.

### Admissions for device complications

Table 4 shows the number of patients with postimplantation stroke and specific device-related coded complications. Due to the lack of present on admission information in HES, figures are presented in two ways, with and without including the index admission. Online supplemental figure A2–A4 shows the time to stroke, which for the majority was within 500 days of implantation.

**Table 4:** Five-year complications following LVAD implantation

Complication	Not including index admission		Including index admission	
	Number (number of deaths if stroke)	Rate as %	Number (number of deaths if stroke)	Rate as %
Haemorrhagic stroke	5 (3)	3.2	11 (8)	7.0
Cerebral infarction	13 (5)	8.3	22 (11)	14.0
Haemorrhagic stroke or cerebral infarction*	17 (8)	10.8	32 (19)	20.4
Any stroke (ICD-10 I60-I64)	24 (14)	15.3	41 (27)	26.1
Infection from device	33	21.0	37	23.6
New LVAD implanted	21	13.4	21	13.4

*One additional patient had an admission with ICD-10 I64X (stroke not specified as haemorrhage or infarction).

LVAD, left ventricular assist device.

## Discussion

### Summary of findings

Despite being young compared with patients in some previous studies, our set of unselected patients with LVAD were multimorbid, with high postimplantation morbidity, mortality and use of hospital services. Within 5 years of LVAD implantation, nearly 60% of our study population had died, the majority from noncardiovascular causes, and approximately one in four had a heart transplant. The median time on support was just short of a year, with huge variation. Thirteen per cent of patients had a second LVAD implanted within 5 years of their first. Hospital admissions per person at risk rose to a peak at the month of implantation before falling away and were dominated by unplanned stays of at least one night with a non-HF primary diagnosis, in common with hospitalised patients with HF in general.^17^ After implantation, ED visits fell but outpatient appointments rose. Stroke and infections from the device were common, with around one in four patients affected by each.

### Comparison with previous studies

Comparisons with other studies are complicated by the different types of device, patient selections, treatment goals and follow-up durations. Our study had the longest follow-up. Two years after implantation, our mortality rate of 48% is higher than that of MOMENTUM-3 (Multicenter Study of MagLev Technology in Patients Undergoing Mechanical Circulatory Therapy with HeartMate 3) (25% and 32% for the two trial groups),^18^ the HeartMate II mid-trial (39% and 35%)^11^ and Tsiouris *et al*’s single-institution cohort (29%, although their 4-year rate was 55%, which was slightly higher than ours),^19^ but it is lower than in an older study from 2007 (69%)^20^; our 1-year rate of 40% is higher than that from the European INTERACS trial-based registry, which reported rates of 24% during the trial and 15% post-trial.^21^ A recent report from the ELEVATE Registry (Evaluating the HeartMate 3 with Full MagLev Technology in a Post-Market Approval Setting), designed to study long-term outcomes with the more modern Heartmate 3 devices, gave a 2-year death rate of just 17%.^22^ There is a trend towards less-invasive nonsternotomy approaches. For example, the LATERAL single-arm trial evaluated the HeartWare centrifugal-flow ventricular assist device system reported that 88% of its 144 patients were alive and free from disabling stroke, transplantation and explantation at 6 months.^23^

Complications of LVAD therapy include bleeding, infection, pump thrombosis, right HF, device malfunction and stroke.^24^ Bleeding is the most common but is not well captured by ICD-10. We report 5-year rates of device-related infection of 23.6% and any stroke of 26.1%. A 2009 trial^11^ comparing continuous flow with pulsatile flow LVADs found the former to have lower adverse event rates, including LVAD-related infection of 0.48 per patient-year (therefore, 48%, around two times our rate despite only having a 2-year follow-up) and a stroke rate of 0.13 per patient-year (therefore, 13%, lower than our rate at 2 years). A 2011 one-institution series found a drive-line or pocket infection rate of 0.72 per patient-year in their 86 patients with continuous flow devices.^25^ The MOMENTUM-3 trial’s 2-year stroke rates were 10% for the HeartMate 3% and 19% for the HeartMate II devices.^18^

### Strengths and limitations

Our study benefits from a 5-year follow-up period with national data and capture of all deaths occurring in England by linkage to the Office of National Statistics death register. A British Heart Foundation report^26^ and NHS Blood and Transplant report^27^ analysed LVADs or ECMOs during the 11-year period from April 2007 to March 2017; for the last 3 years, there were 82 long-term LVADs per year. We had 157 in 2 years, which matches the external source well. The type of LVAD is not recorded in HES. An inevitable downside of our longer follow-up is that we are evaluating older technologies.

We grouped the cause of death broadly to reduce misclassification, which can be a problem with death certificates. The primary diagnosis and procedure fields are known to be highly accurate,^28^ giving confidence in the numbers of LVADs (as noted above) and heart transplants. Secondary diagnosis coding is less accurate and subjected to variations by hospital. ICD-10 coding limitations restricted what we were able to report regarding disease severity and complications. Bleeding is poorly captured and, in our experience, blood transfusion during or after surgery often unrecorded. For infections, we used the device-specific ICD-10 T code rather than a broader set of infection codes, which would have captured more but at the cost of being less likely to relate specifically to the device. For stroke, we used several definitions. As HES lacks present on admission information, we ran the complication analysis with and without the index admission. As with infections, stroke codes in the index admission could sometimes relate to previous events rather than to the LVAD.

## Conclusions

Mortality is high and hospital use and complications are common in the 5 years following LVAD implantation. Administrative data provide important information on resource use in this patient group. Such data have national coverage and, whereas registries typically publish annual reports,^29 30^ HES is available to researchers only 3 months behind real time and can, therefore, give more timely estimates of hospital use.

## Data Availability

Data may be obtained from a third party and are not publicly available. Deidentified Hospital Episode Statistics data as used in this study are available from NHS Digital for authorised users.
