# Supplementary material for: Five-year outcomes following left ventricular assist device implantation in England
Source: Open Heart. 2021 May 11;8(1):e001658. doi: 10.1136/openhrt-2021-001658 (PMC8117985; doi:10.1136/openhrt-2021-001658)
Supplement: Supplementary data [file openhrt-2021-001658supp001.pdf]

Appendix. Supplementary materials

FIVE-YEAR SURVIVAL AND USE OF HOSPITAL SERVICES FOLLOWING LEFT VENTRICULAR ASSIST DEVICE IMPLANTATION IN ENGLAND

Alex Bottle, Puji Faitna, Paul Aylin, Martin R Cowie

Contents

Table A1. ICD10 codes for adverse events. Page 1

Figure A1. Time between LVAD implantation and admission for device-related infection. Page 2

Figure A2. Time between LVAD implantation and admission for any stroke (ICD10 I60-I64). Page 3

Figure A3. Time between LVAD implantation and admission for haemorrhagic stroke. Page 4

Figure A4. Time between LVAD implantation and admission for infarction stroke. Page 5

Table A2. NHS reference costs for total inpatient and day case admissions, ED visits not ending in admission, and outpatient appointments in the 12 months before and the 12 months after the index month (index month not included). Page 6

Table A1. ICD10 codes for adverse events

ICD-10	Description
I60	Subarachnoid haemorrhage
I61	Intracerebral haemorrhage
I62	Other nontraumatic intracranial haemorrhage
I63	Cerebral infarction
I64	Stroke, not specified as haemorrhage or infarction
T821	Mechanical complication of cardiac electronic device
T827	Infection and inflammatory reaction due to other cardiac and vascular devices, implants and grafts (excluding cardiac valve prostheses)
T828	Other complications of cardiac and prosthetic vascular devices, implants and grafts (including embolism, pain etc.)
T829	Unspecified complications of cardiac and prosthetic vascular devices, implants and grafts

Bottle A, *et al. Open Heart* 2021; 8:e001658. doi: 10.1136/openhrt-2021-001658

The next four plots are of time to admission for infection and stroke. A few events occurred during the index admission itself.

Figure A1. Time between LVAD implantation and admission for device-related infection

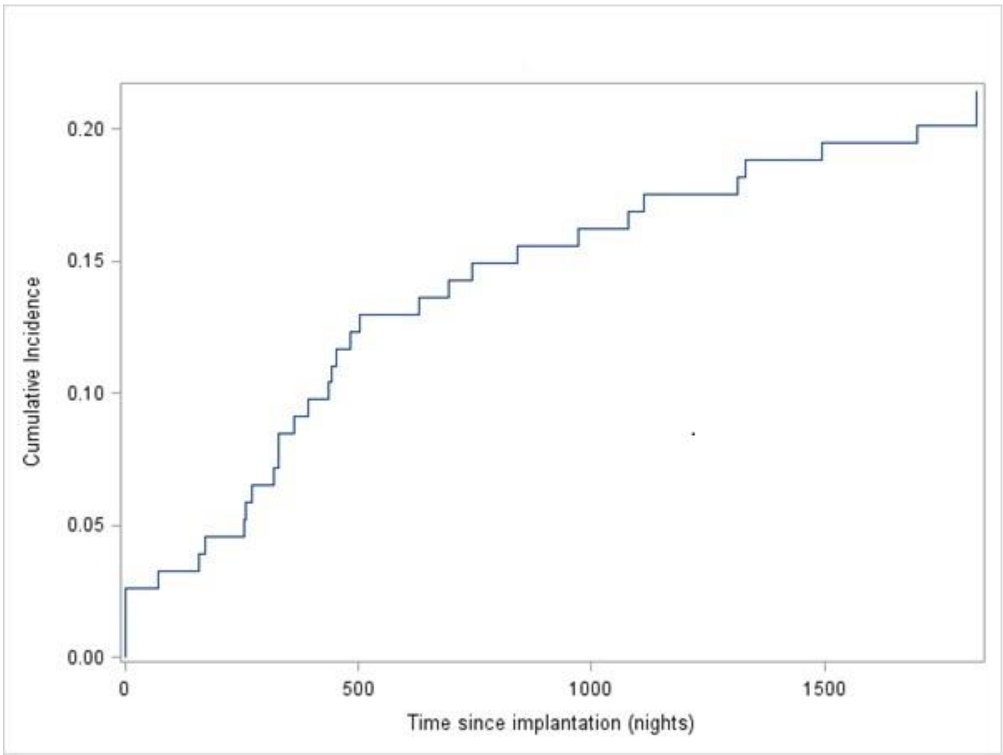

Figure A2. Time between LVAD implantation and admission for any stroke (ICD10 I60-I64)

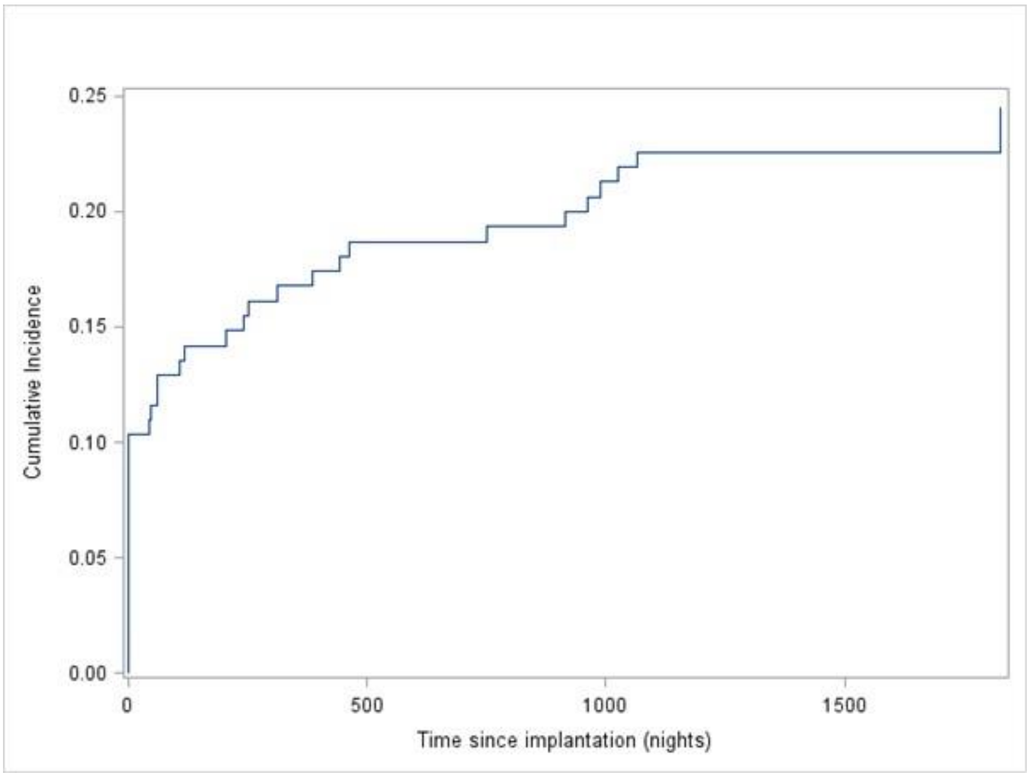

Figure A3. Time between LVAD implantation and admission for haemorrhagic stroke

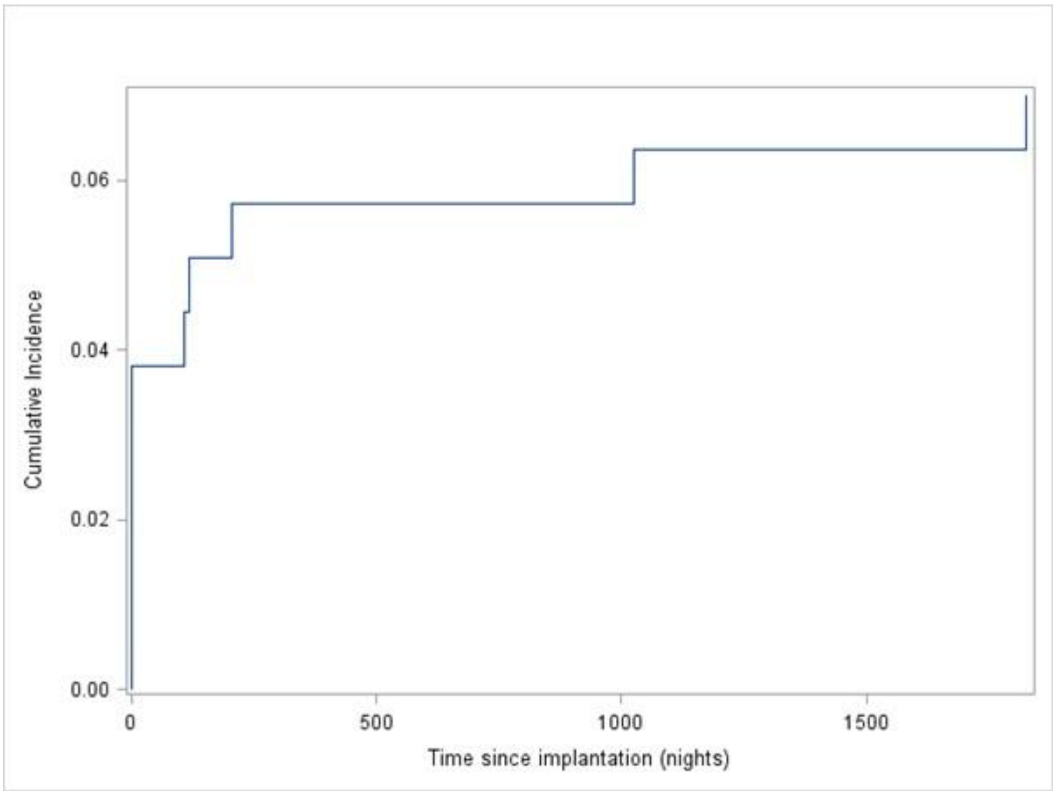

Figure A4. Time between LVAD implantation and admission for infarction stroke

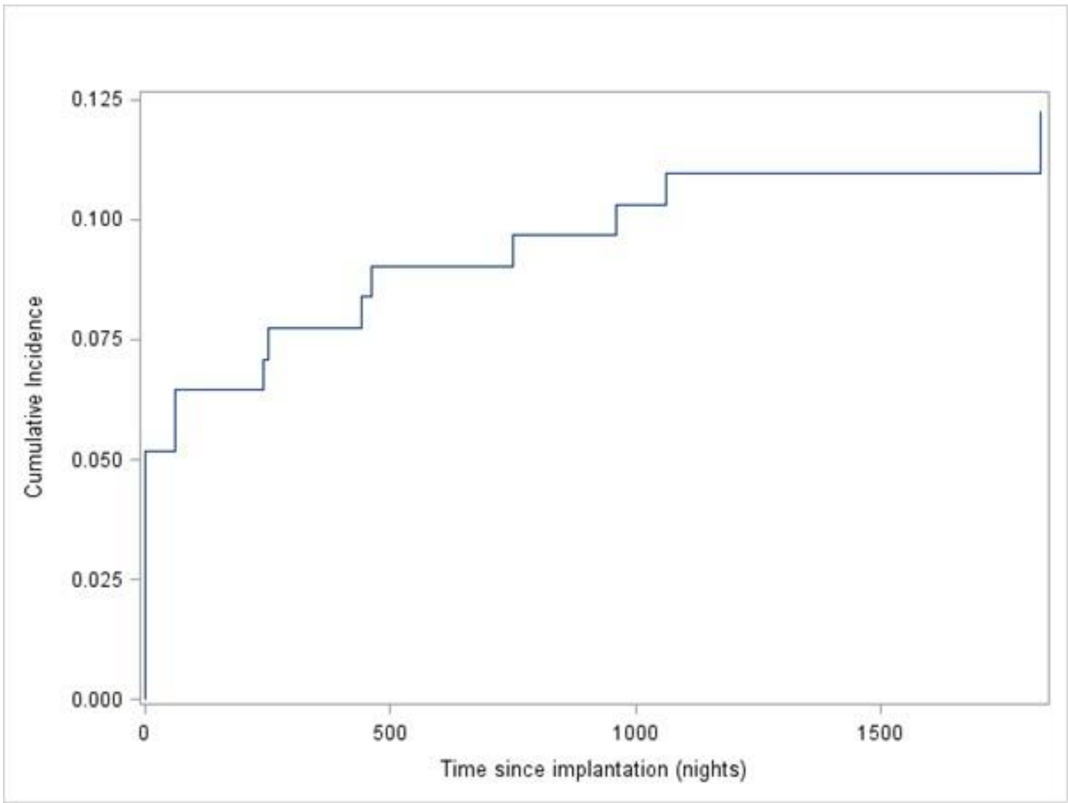

Table A2. NHS reference costs for total inpatient and day case admissions, ED visits not ending in admission, and outpatient appointments in the 12 months before and the 12 months after the index month (index month not included)

	12m before index	12m after index
All admissions	421	344
Total cost (£)	985,267	803,027
Admissions per patient at risk	0.223	0.271
Mean cost per patient at risk (£)	522.97	632.80
Total ED visits not ending in admission	80	53
Total cost (£)	8,731	6,006
Mean cost per patient at risk (£)	6.50	5.82
Total outpatient department appointments	1,356	2,057
Total cost (£)	142,702	222,405
Mean cost per patient at risk (£)	79.28	177.36

Bottle A, *et al. Open Heart* 2021; 8:e001658. doi: 10.1136/openhrt-2021-001658
